# Proteomics of stress responses in wheat and barley—search for potential protein markers of stress tolerance

**DOI:** 10.3389/fpls.2014.00711

**Published:** 2014-12-11

**Authors:** Klára Kosová, Pavel Vítámvás, Ilja T. Prášil

**Affiliations:** Laboratory of Plant Stress Biology and Biotechnology, Division of Crop Genetics and Breeding, Department of Plant Genetics, Breeding and Product Quality, Crop Research InstitutePrague, Czech Republic

**Keywords:** proteome, barley, wheat, abiotic stress factors, biotic stress factors, protein markers

## Abstract

Wheat (*Triticum aestivum; T. durum*) and barley (*Hordeum vulgare*) agricultural production is severely limited by various abiotic and biotic stress factors. Proteins are directly involved in plant stress response so it is important to study proteome changes under various stress conditions. Generally, both abiotic and biotic stress factors induce profound alterations in protein network covering signaling, energy metabolism (glycolysis, Krebs cycle, ATP biosynthesis, photosynthesis), storage proteins, protein metabolism, several other biosynthetic pathways (e.g., S-adenosylmethionine metabolism, lignin metabolism), transport proteins, proteins involved in protein folding and chaperone activities, other protective proteins (LEA, PR proteins), ROS scavenging enzymes as well as proteins affecting regulation of plant growth and development. Proteins which have been reported to reveal significant differences in their relative abundance or posttranslational modifications between wheat, barley or related species genotypes under stress conditions are listed and their potential role in underlying the differential stress response is discussed. In conclusion, potential future roles of the results of proteomic studies in practical applications such as breeding for an enhanced stress tolerance and the possibilities to test and use protein markers in the breeding are suggested.

## Introduction

Wheat (*Triticum aestivum*; *T. durum*) and barley (*Hordeum vulgare*) represent major cereal crops grown in temperate climate areas. Cereal agricultural production is limited by a wide array of abiotic and biotic stress factors including drought (Cattivelli et al., 2008), cold (Thomashow, 1999; Kosová et al., 2008a), heat, salinity (Munns, 2005; Kosová et al., 2013a), imbalances in mineral nutrition, viral (Kosová et al., 2008b) and fungal pathogens such as *Fusarium* (Kosová et al., 2009; Yang et al., 2010a,b), leaf rust (*Puccinia triticina*; Rampitsch et al., 2006), blotch (*Septoria tritici*; Yang et al., 2013) and others, often acting in combinations under field conditions (Mittler, 2006). Proteome plays an important role in stress response since proteins are directly involved in several processes aimed at an enhancement of stress tolerance being “closer to phenotype” than transcripts.

During the past decades, the boom of high-throughput proteomics techniques has enabled the researchers to study plant proteome responses to various factors including stresses in a complex way. Despite numerous studies reporting identifications of a few thousand of proteins in plant samples, a complete description of plant proteome in a given tissue, developmental phase and environmental conditions still remains a great challenge (Jorrin-Novo et al., 2009).

Both abiotic and biotic stresses induce profound changes in plant proteomes aimed at an adjustment of metabolism to altered environment and an enhancement of plant stress tolerance. Plant stress response is a dynamic process and several phases with a unique proteome composition could be distinguished (Levitt, 1980; Larcher, 2003). Reviews on plant proteome responses to abiotic stresses (Kosová et al., 2011; Hossain et al., 2012) and pathogens (Sergeant and Renaut, 2010; Gonzalez-Fernandez and Jorrin-Novo, 2012) provide important overviews; however, several novel studies were published recently (Table 1).

**Table 1:** **A list of proteomic studies focused on abiotic and biotic stress responses in wheat (*Triticum aestivum; T. durum*), barley (*Hordeum vulgare*), and related species**.

**Plant material**	**Treatment**	**Methods**	**Major differentially-abundant proteins (DAP)**	**References**
**LOW TEMPERATURE (COLD, FROST)**				
Winter wheats (*Triticum aestivum*) Mironovskaya 808 (T) and Bezostaya 1(t)–leaf	2°C (21 days)	0.1 M Tris-HCl, pH 9; 2DE LC-MS/MS	Up: WCS120, WCS19, COR14a–higher levels in T	Vítámvás et al., 2007
Winter wheats Norstar (T) and Azar2 (t)–leaf	2°C (0, 14, 28, 42, 56 days)	2DE MALDI-TOF/TOF	Up: COR/LEA (WCOR14a, WRAB17, WRAB18); Cu/Zn-SOD, 2-2-Cys Prx, GST–higher levels in T	Sarhadi et al., 2010
Winter wheat Cheyenne (T)–leaf	4°C (63 days)	TCA/acetone; 2DE MS/MS	Up: WCOR18, WRAB17, WCOR615; VER2, glycine-rich RNA binding protein	Rinalducci et al., 2011a
Spring wheat Kohdasht (S)–leaf	4°C (42 days) Control: 20°C (42 days)	2DE nanoLC-MS/MS	Up: APX, DHAR, COR/LEA, cysteine proteinase, proteasome subunit α	Rinalducci et al., 2011b
			Down: glycolysis (GAPDH, TPI), Krebs cycle enzymes (MDH), ATP synthase β, ε; PSII subunits	
Winter wheats Mironovskaya 808 (T) and Bezostaya 1 (t)–crown	6°C (0, 3, 21, 84 days)	TCA/acetone/phenol; 2D-DIGE MALDI-TOF/TOF	298 DAP (202 identified)	Vítámvás et al., 2012
			Up: 3-PGK, TPI, PGM, ENO; HSP70; MDAR, DHAR, GPX, GST	
			Down: ALDO, GAPDH; SUS1, UDP-glucose pyrophosphorylase; 14-3-3; HSP90; APX	
			Vernalization: Chopper chaperone Genotypic differences: MDH, legumin-like protein–higher in T than t	
Winter wheat Samanta (T), spring wheat Sandra (S)–crown	4°C (0, 3, 21 days)	TCA/acetone/phenol; 2D-DIGE MALDI-TOF/TOF	58 DAP (36 identified)	Kosová et al., 2013b
			Up: GAPDH, β subunit ATP synthase, CPN60-α, CPN60-β	
			Down: FRK-2, SUS1, 11S seed storage protein	
			Genotypic differences: methionine synthase, eIF3, eIF5A2–higher in T; VER2, sGRP–higher in S	
Winter wheats–Shixin 828 (T), Shiluan 02-1 (t)–leaf	−8°C (5 h)	TCA/acetone; 2DE MALDI-TOF/TOF	RubisCO LSU and SSU; α and β subunit ATP synthase; V-ATPase; MDH;	Xu et al., 2013
			Genotypic differences: RubisCO LSU and SSU, PRK; Mn-SOD–higher in T than t	
Wild wheat (*Triticum urartu*)–leaf	4–6°C (28 days) followed by −2°C (12 h)	TCA/acetone; 2DE MALDI-TOF/TOF	34 identified proteins–25 up- and 9 down-regulated	Gharechahi et al., 2014
			Up: LEA-III, WCOR14, PR4; OEE1, chloroplastic ribosomal protein L12	
			Down: RubisCO SSU	
Barley (*Hordeum vulgare*) Winter barley Luxor (T)–crown, leaf	3°C (0, 1, 21 days), −3°C (1 day)	TCA/acetone/phenol; 2D-DIGE MALDI-TOF	Up: HSP70; OEE1 (PsbO),	Hlaváèková et al., 2013
			Down: eEF-Tu; GS1 and 2; UDP-glucose 6-dehydrogenase	
			Both leaf and crown: AAA ATPase,	
**HEAT**				
			V-ATPase; eEF-Tu, CPN60, 60S and 40S ribosomal proteins; GS	
Common wheat–Fang (T), Wyuna (S)–grain endosperm	40/25°C (day/night)–15, 16, 17 days post-anthesis	TCA/acetone 2DE MALDI-TOF; MS/MS Q-TOF	Genotypic differences: Seven small HSP (16.9 kD class I HSP) proteins unique to T	Skylas et al., 2002
Common wheat–Thésée–grain	34/10°C (day/night)–697 and 763°C d (degree–days)	Sodium-phosphate buffer; 2 DE MALDI-TOF	42 identified proteins	Majoul et al., 2004
			Up: 20 kD sHSP, 17 kD class II HSP; HSP82 (HSP90 family); eEF-Tu, V-ATPase subunit E	
			Down: starch biosynthesis enzymes granule-bound starch synthase, glucose-1-phosphate adenyltransferase; β-amylase; β subunit ATP synthase	
**DROUGHT**				
Common wheat–spring wheats Arvand, Khazar-1, Kelk Afghani–grain	Field conditions (Azarbayjan) plus artificial irrigation	2DE MALDI-TOF/TOF	121 (57 identified) Up: Trx *h*, 1-Cys peroxiredoxin, GST; PDI; LEA, sHSP17, HSP70	Hajheidari et al., 2007
Australian wheats Kukri (S), Excalibur, RAC875 (T)–leaf	Water witholding until leaf wilting in Kukri (S)–14, 24 days, and rewatering (25 days)	TCA; nanoLC-MS/MS iTRAQ 8plex	1299 identified proteins Increase in ROS metabolism-associated proteins (CAT, Cu/Zn-SOD, Mn-SOD), decrease in photosynthesis and Calvin cycle-related proteins (RubisCO; PSI subunit VII PsaC)	Ford et al., 2011
			Genotypic differences: COR410–higher increase in T than S	
Common wheat cv. Nesser (T), Opata M85 (S)–root	21°C; 40 % humidity–combined effect of drought and ABA (100 μM)	nanoLC-MS/MS iTRAQ	1656 identified proteins	Alvarez et al., 2014
			805 ABA-responsive proteins: LEA, protein phosphatases PP2C;	
			Genotypic differences: HSP70, HSP90; 14-3-3, G-proteins; V-ATPase–higher in T; β-expansin, porins–higher in S	
Durum wheat cv. Ofanto–leaf	70 % FWC for 7 days (control); 57 % FWC for 7 days (stress)	175 mM Tris-HCl, pH 8.8, TCA-acetone; 2DE MALDI-TOF	36 identified proteins	Caruso et al., 2009
			Up: carbonic anhydrase, RubisCO LSU	
			Down: RubisCO SSU, Calvin cycle enzymes (ALDO, PRK); ATP synthase CF1 α; plastidic GS2a,b,c	
Durum wheat cv. Kiziltan (S), emmer (*T. dicoccoides*) lines TR39477, TTD22 (T)–leaf	9 days without watering	2DE nanoLC-ESI-MS/MS	75 identified proteins, 11 candidates for drought tolerance	Budak et al., 2013
			Genotypic differences: TPI, ATP synthase CF1 (efficient carbohydrate metabolism and ATP production)–higher in T; β-1,3-glucanase, β-1,4-glucanase, XET (cell wall remodeling for osmotic adjustment and energy source); methionine synthase–higher in S	
Barley cv. Basrah (T) and Golden Promise (S)–leaf, root	7 days without watering Control: 80 % RWC; Drought: 70 % RWC (T), 60 % RWC (S)	10 mM PBS, TCA-acetone; 2D-DIGE MALDI-TOF	Identified proteins: 24 (leaf), 45 (root)	Wendelboe-Nelson and Morris, 2012
			Up: ABA-induced protein r40c1, small G-protein Rab2, Myb-like protein, 14-3-3 protein	
			Down: GST, GPX	
			Genotypic differences: Enhanced regulation of ROS (APX, CAT, LOX, class III POX) and protein folding in T than in S	
Barley–8 Egyptian accessions, 2 selected for proteome analysis 15141 (T), 15163 (S)–leaf	24°C; 70 % FWC (control); 5 days at 10 % FWC (stress)	TCA/acetone; 2D-DIGE MALDI-TOF	Up: PDI, Hsp90, Hsp100 (Clp protease), chloroplastic ATP synthase CF1 α;	Ashoub et al., 2013
			Genotypic differences: PPDK, Hsp70, zinc metalloprotease–higher in T than S; proteins involved in osmolyte biosynthesis (betaine aldehyde dehydrogenase, methionine synthase, SUS1)–higher in S than T	
Barley cv. Golden Promise–leaf	Ca 100% FWC (control); 25 % FWC (stress)–28 days–combined effect of drought and *Piriformospora indica*	TCA/acetone; 2DE MALDI-TOF/TOF	45 identified proteins	Ghabooli et al., 2013
			Up: RubisCO activase A, RubisCO SSU, CCOMT	
			Down: PRK, ACP	
			Effect of *P. indica*: RubisCO SSU, PSI Fe-S center, chl *a*/*b* binding protein; CCOMT, APX, 30S ribosomal protein 3, V-ATPase, 2-Cys Prx–higher in inoculated than control plants under stress	
**OSMOTIC STRESS (PEG-6000)**				
Common wheat cv. Yumai 34–leaf	Hoagland solution, 15% PEG-6000 (3 days); 0.5 mM SA pretreatment (3 days)	TCA/acetone; 2DE MALDI-TOF/TOF	82 (76 identified proteins), of which 35 SA-responsive proteins	Kang et al., 2012
			Up: 14-3-3; APX, GST,	
			SA-responsive proteins: GS1c, GST1, PDI; ATP synthase CF1 α,β	
Common wheat–spring wheats Abbondanza (T), Qingchun 38 (S)–leaf	PEG-6000 (−1 MPa; 72 h), recovery (24 h)	TCA/acetone; 2DE MALDI-TOF/TOF	38 (35 identified proteins)	Ye et al., 2013
			Up: GAPDH B; 26S proteasome, V-ATPase A	
			Down: RubisCO LSU and SSU, GAPDH, TPI, AGPase (starch biosynthesis)	
			Genotypic differences: Less PEG-affected proteins in T than S	
Common wheat cv. Hanxuan 10 (T) and Ningchun 47 (t)–seedling leaf	Hoagland solution, 20% PEG-6000 (−075 MPa) for 48 h	TCA/acetone/phenol; phosphopeptide enrichment via TiO_2_ microcolumns; LC-MS/MS	173 (T) and 251 (t) phosphoproteins identified	Zhang et al. (2014)
			Phosphoproteins identified: signaling (SnRK2 kinase, protein phosphatase 2C, CDPK, calmodulin 2-2); transport (AQP, MSSP2; H^+^-ATPase); LEA proteins (WCOR719, WCOR825, WRAB17)	
**SALINITY**				
Common wheat (*T. aestivum*) Jinan 177 (S), *T. aestivum* × *Thinopyrum ponticum* Shanrong 3 (T)–seedling root	½Hoagland solution, 200 mM NaCl (24 h)	TCA/acetone; 2DE MALDI-TOF, MALDI-TOF/TOF	114 (110 identified–49 salt-responsive, 34 genotypic differences)	Wang et al., 2008
			Up: 14-3-3;	
			Down: tubulin α-3	
			Genotypic differences: DEAD-box RNA helicase, DWARF3 (GA biosynthesis), eIF5A2, V-ATPase subunit E–higher in T; G-protein β subunit, ethylene receptor ETR1–higher in S	
Common wheat cv. Calingiri, Janz (S), Wyalkatchem (T)–shoot mitochondrial fraction	200 mM NaCl (increase per 50 mM NaCl/days)	Isolation: PVP gradient; acetone extraction; 2D-DIGE LC-MS/MS	192 DAP (68 identified)	Jacoby et al., 2010
			Up: AOX, Mn-SOD, VDAC	
			Down: CS, NDPK, outer mitochndrial membrane porin	
			Genotypic differences: AOX, Mn-SOD–higher in T	
Common wheat cv. Keumgang–leaf chloroplast fraction	150 mM NaCl (1, 2, 3 days)	Isolation: Percoll gradient; TCA/acetone; 2DE LTQ-FTICR-MS	100 DAP (65 identified)	Kamal et al., 2012
			Up: RubisCO, GAPDH, GDH, PDX1.2 and PDX1.3	
			Down: ATP synthase α,β,γ; V-type proton ATPase	
Common wheat (*T. aestivum*) cv. Chinese Spring (S), *T. aestivum* × *Lophopyrum elongatum* amphiploid (T)–mitochondrial fraction (shoot, root)	200 mM NaCl (increase per 50 mM NaCl/days)	100% acetone (leaf), TCA/acetone (root); 2D-DIGE MALDI-TOF/TOF; HPLC Q-TOF MS/MS (peptide fingerprinting–genotypic differences)	55 root, 15 shoot differentially abundant proteins	Jacoby et al., 2013
			Organ-specific differences: aspartate aminotransferase, GDH (up in shoot, down in root)	
			Genotypic differences: Mn-SOD, MDH, aconitase, SHMT, β-CAS–higher in T	
Durum wheat (*T. durum*) cv. Ofanto -leaf	100 mM NaCl (2 days)	TCA/acetone 2DE MALDI-TOF	38 identified proteins	Caruso et al., 2008
			Up (28): TPI; CPN60-β, RubisCO activase, carbonic anhydrase; osmolyte biosynthesis-related enzymes (glycine dehydrogenase, SAMS); COR; Cu/Zn-SOD	
			Down (10): ALDO, PGK, RubisCO SSU, OEE1 precursor, β-glucosidase, ATP synthase CF1 α	
Durum wheat cv. Waha–seed embryo and surrounding tissue	250 mM NaCl (42 h)–AsA priming (0.5 mM)	KCl (100 mM), acetone/nanoHPLC-MS	697 identified proteins–proteins involved in energy metabolism, protein metabolism, disease/defense, protein destination, storage–a positive effect of AsA priming on mitigation of salinity stress	Fercha et al., 2013, 2014
Barley cv. OUK305 (T), OUI743 (S)–root	200 mM NaCl (5 days)	40 mM Tris, 8 M urea, 4% CHAPS, 0.2% Bio-Lyte; 2DE nanoLC-ESI-MS/MS	6 differentially abundant proteins CCOMT, DHAR, GST (2 spots), POX, PR10–higher in T than S	Sugimoto and Takeda, 2009
Barley cv. Morex (T), Steptoe (S)–root	100, 150 mM NaCl (13 days)	TCA/acetone; 2DE MALDI-TOF; nanoLC-ESI-Q-TOF MS/MS	39 differentially abundant proteins	Witzel et al., 2009
			Up: LOX1, POX, SAMS, β-1,3-glucanase	
			Down: IDI1, IDI2, IDS2, IDS3,	
			Genotypic differences: class III POX, SAMS–higher in T; APX, MDAR–higher in S	
Barley cv. Afzal (T), L-527 (S)–leaf	300 mM NaCl (increase per 50 mM NaCl/days) 24 h	TCA/acetone; 2DE MALDI-TOF/TOF	117 DAP (22 identified proteins)	Rasoulnia et al., 2011
			Up:, PC, OEE2, PSI subunit VII (PsaC), PRK; 2-Cys Prx, Trx, GST, SOD; TPI, FBP ALDO–higher in T than S	
Barley cv. Afzal (T), L-527 (S)–leaf	300 mM NaCl (increase per 50 mM NaCl/days)–21 days	TCA/acetone; 2DE MALDI-TOF/TOF	44 DAP Up (43): RubisCO LSU, SSU, RubisCO activase, OEE2; NDPK; GLP; profilin; ribosomal protein L12, 30S ribosomal protein S1; translationally-controlled tumor protein homolog	Fatehi et al., 2012
			Genotypic differences: DHAR, Trx–higher in S	
Barley cv. Morex (T), Steptoe (S)–root	100, 150 mM NaCl (0, 1, 4, 7, 10 days)	TCA/acetone; 2DE MALDI-TOF; nanoLC-ESI-Q-TOF MS/MS	91 DAP (74 identified proteins)	Witzel et al., 2014
			Genotypic differences: GLP3-7, GLP12, β-1,3-glucanase, ATP synthase CF1 β–higher in T; GLP5a, PR17–higher in S	
**COMBINED STRESS**				
Osmotic stress or salinity–common wheat (*T. aestivum*) Jinan 177 (S), *T. aestivum* × *Thinopyrum ponticum* Shanrong 3 (T)–root, leaf	½Hoagland solution 18% PEG-6000 or 200 mM NaCl (24 h)	TCA/acetone; 2DE MALDI-TOF/TOF	93 (root), 65 (leaf) differentially abundant proteins; 34 (root), 6 (leaf)–genotypic differences	Peng et al., 2009
			PEG: 38 root, 39 leaf;	
			Salinity: 52 root, 52 leaf proteins	
			PEG-specific proteins: ribosomal protein S8 (↓)	
			Salt-specific proteins: importin α 1b (root),	
			Genotypic diffrences: chl *a*/*b* binding apoprotein CP24 precursor, DWARF3–higher in T	
Drought and heat Barley–Syrian landrace Arta (T), Australian cv. Keel (T)–leaf (heading stage)	Drought: 50% FWC (control), 15% FWC (stress) for 3 days Heat: 36°C (4 h)	TCA/acetone; 2DE, 2D-DIGE MALDI-TOF/TOF	99 DAP Heat–up: FBP ALDO, chaperones, proteases, eEF-G, eIF4A, RubisCO activase B	Rollins et al., 2013
			Genotypic differences (14 proteins): photosynthesis-related proteins (LHCII type III Lhcb3, OEE1 PsbO, RubisCO activase B)–higher in Keel than Arta	
Drought or waterlogging and cold–winter common wheat cv. Yannong 19–leaf	Drought + LT: 35% FWC (7 days) Waterlogging + LT (7 days)	TCA/acetone; 2DE MALDI-TOF/TOF	32 identified proteins	Li et al., 2014
			Up: DHAR, GR; Hsp70;	
			Down: C metabolism-related proteins (glycolysis, TCA, Krebs cycle), RubisCO activase A, ATP synthase CF1 α,β	
**IMBALANCES IN MINERAL NUTRIENTS**				
**Boron**				
Barley Clipper (S) × Sahara (T) DH lines–leaf, root	1 mM H_3_BO_3_ (S), 5 mM H_3_BO_3_ (T) for 14 days	PBS pH 7.5, TCA/acetone; 2D-nanoLC-MS/MS iTRAQ	138 (leaf), 341 (root) identified proteins	Patterson et al., 2007
			Up: IDS2, IDS3, methyl-thioribose kinase	
			Leaf: PRK, PGK, PGM, ENO, PC, RubisCO activase, eEF1-α,β,γ; eEF-G, eEF-Tu; TLP; Cu/Zn-SOD; 50S ribosomal protein L3; 60S ribosomal protein L1	
			Root: CCOMT, class III POX, chitinase, 26S proteasome, β-1,3-glucanase; ATP synthase CF1 β, IDS2, IDS3; Hsp70; Hsc70; 40S ribosomal protein S5	
**Copper**				
Common wheat cv. Yumai 34–leaf, root	100 μM CuSO_4_ (3 days)	TCA/acetone; 2DE MS/MS	98 DAP [93 identified proteins–43 (leaf), 49 (root)] 36 Cu-responsive proteins	Li et al., 2013
			Leaf: 14-3-3; MDH, TPI; PDI; V-ATPase A; ATP synthase CF1 α; carbonic anhydrase, RubisCO activase, PSI subunit VII (PsaC);	
			Root: 14-3-3, translationally-controlled tumor protein; Hsp70, APX, GST, Cu/Zn-SOD, PR10; TPI, ATP synthase CF1 α; actin 1, tubulin	
**Nitrogen**				
Common wheat cv. Arche, Récital–leaf	2, 8, 20 mg N/plant/d for 60 days	TCA/acetone; 2DE LC-MS/MS	76 DAP (14 identified proteins) FBP ALDO, PGK, PGM, ENO2, MDH; RubisCO activase A, OEE1 (PsbO); 2-Cys Prx	Bahrman et al., 2004
**PATHOGENS**				
***Fusarium culmorum* and *F. graminearum* (teleomorph *Gibberella zeae*)**				
Barley cv. Scarlett (S)–young spikelet	artificial inoculation *F. Graminearum*–3 days	Acetone; 2DE MALDI-TOF	51 DAP (50 identified)	Yang et al., 2010a
			Up: PR proteins (PR-1,2,3,5,9,15); proteolytic fragments of β-amylase induced by pathogen	
Barley cv. Scarlett (S)–mature grain	artificial inoculation *F. Graminearum*–72 h; 15, 100 kg ha^−1^ N	5 mM Tris-HCl pH 7.5 (water soluble proteins), 2DE MS/MS	Up: 80 proteins (serpin, protease inhibitors CI-1A, CI-1B)	Yang et al., 2010b
			Down: 108 proteins (albumins) 65 proteolytic fragments (albumins, serpin, protease inhibitors) 9 proteins of *F. graminearum* (peptidyl-prolyl cis-trans isomerase, Cu/Zn-SOD, L-xylulose reductase) Positive effect of increased N on plant resistance	
Naked barley (*Hordeum vulgare* ssp. *nudum*)–mature grain	1.2 mg/kg DON (artificial inoculation *F. culmorum* and *F. graminearum*)	50 mM Tris-HCl pH 7.4; NEPHGE 2-DE MALDI-TOF nanoLC-MS/MS	11 identified proteins	Eggert and Pawelzik, 2011
			Up: DNA-dependent RNA-polymerase; Dof zinc-finger protein, NBS-LRR (transcription regulation); serpin (3 spots; serine protease inhibitor);	
			Down: ADP-glucose pyrophosphorylase	
Emmer (*Triticum dicoccum*)–mature grain	10 mg/kg DON (artificial inoculation)	TCA/acetone; 2DE nanoLC-MS/MS	10 identified proteins	Eggert et al., 2011
			Up: serpin (serine protease inhibitor), TLP; β-amylase, globulin	
			Down: POX, Prx; glycosyltransferase; chitinase; α-gliadin	
***Puccinia triticina***				
Common wheat cv. Thatcher (S), NIL Thatcher*Lr1* (T)–leaf	3, 6, 9 days after artificial infection	TCA/acetone; 2DE qTOF-MS/MS	32 identified proteins (S); T showed no reproducible response	Rampitsch et al., 2006
			Up: eEF1-β, eIF5A2, 20S proteasome subunit α-1, ribosomal protein P0; TPI; dihydrolipoamide acetyl transferase; α-tubulin; Hsp70, CPN60; ATP synthase CF1 β; peptidyl-prolyl *cis-trans* isomerase	
***Septoria tritici* (teleomorph *Mycosphaerella graminicola*)**				
Common wheat cv. Sevin (S), Stakado (T)–leaf	3, 7, 11 days after artificial infection	Phenol extraction; phosphoprotein separation: Poros Oligo R3 micro-column; LC-MS/MS	Plant: Phosphoproteins (70 in T, 60 in S)–signaling (CDPK, MAPK); transport (PIP ATPase)–higher in T than S	Yang et al., 2013
			Pathogen: 31 proteins, 5 phosphoproteins (G-proteins, 14-3-3; Ras GTPase; ABC transporter)	

*Abbreviations: 2Cys-Prx, 2-cysteine peroxiredoxin; 2DE, two-dimensional electrophoresis; 2D-DIGE, two-dimensional differential in-gel electrophoresis; β-CAS, β-cyanoalanine synthase; ABA, abscisic acid; ACP, acyl carrier protein; AGPase, ADP glucose pyrophosphorylase; AOX, alternative oxidase; APX, ascorbate peroxidase; AQP, aquaporin; AsA, ascorbic acid; CCOMT, caffeoyl-coenzyme A O-methyltransferase; COR, Cold-regulated (protein); CPN, chaperonin; CS, cysteine synthase; CDPK, calcium-dependent protein kinase; DAP, differentially abundant proteins; DH, double haploid (line); DHAR, dehydroascorbate reductase; DON, deoxynivalenol; ENO, enolase; FBP ALDO, fructose-1,6-bisphosphate aldolase; FWC, field water capacity; GAPDH, glyceraldehyde-3-phosphate dehydrogenase; GAPDH B, glyceraldehyde-3-phosphate dehydrogenase B form; GDH, glutamate dehydrogenase; GLP, germin-like protein; GPX, glutathione peroxidase; GS, glutamine synthetase; GST, glutathione S-transferase; HPLC, high performance liquid chromatography; Hsc70, heat shock cognate protein 70; iTRAQ, isobaric tag for relative and absolute quantification; LC, liquid chromatography; LEA, Late embryogenesis-abundant (protein); LOX, lipoxygenase; LTQ-FTICR, linear quadruple trap-Fourier transform ion cyclotron resonance; MALDI-TOF/TOF, matrix-assisted laser desorption ionization time-of-flight/time-of-flight (spectrometry); MAPK, mitogen-activated protein kinase; MDAR, monodehydroascorbate reductase; MDH, malate dehydrogenase; MS, mass spectrometry; MSSP2, monosaccharide sensing protein 2; NBS-LRR, nucleotide-binding site leucine-rich repeat protein; NEPHGE, non-equilibrium pH gel electrophoresis; NDPK, nucleoside diphosphate kinase; NIL, near-isogenic line; OEE, oxygen evolving enhancer (protein); PBS, phosphate buffer saline; PC, plastocyanin; PDI, protein disulfide isomerase; PDX, pyridoxal biosynthesis protein; PEG, polyethylene glycol; PGK, phosphoglycerokinase; PGM, phosphoglyceromutase; POX, peroxidase; PPDK, pyruvate phosphate dikinase; PRK, phosphoribulokinase; Prx, peroxiredoxin; PS, photosystem; PVP, polyvinyl pyrrolidone; qTOF, quadrupole time-of-flight; RubisCO, ribulose-1,5-bisphosphate carboxylase/oxygenase; RubisCO LSU, RubisCO large subunit; RubisCO SSU, RubisCO small subunit; RWC, relative water content; S, sensitive (genotype); SA, salicylic acid; SHMT, serine hydroxymethyltransferase; SnRK, sucrose non-fermenting-related protein kinase; SOD, superoxide dismutase; SUS1, sucrose synthase 1; T, tolerant (genotype); t, genotype less tolerant than T; TCA, trichloroacetic acid; TLP, thaumatin-like protein; TPI, triose phosphate isomerase; Trx, thioredoxin; V-ATPase, vacuolar ATPase; VDAC, voltage-dependent anion channel; WCS, Wheat Cold-specific (protein); WRAB, Wheat responsive-to-ABA (protein); XET, xyloglucan endo-transglycosylase*.

Most proteomic papers aimed at an investigation of plant stress responses are comparative studies that are based on comparison of proteome composition in stressed plants vs. control ones, and also in differentially-tolerant genotypes exposed to stress. Moreover, studies on the roles of subcellular proteomes such as chloroplast (Kamal et al., 2012) and mitochondrial (Jacoby et al., 2010, 2013) proteomes as well as posttranslational modifications (PTMs) such as phosphoproteomics (Yang et al., 2013; Zhang et al., 2014) in wheat exposed to stress have been published recently. Considering the increasing amount of proteomic data, it is arising necessary to mine the data published in various proteomic studies in order to identify key proteins involved in plant responses to a wide array of stress factors (dehydrative stresses—drought, osmotic stress, salinity, frost, heat) as well as proteins induced only at specific stress conditions (e.g., phytochelatins and heavy metal stress). An attempt has already been published regarding proteomic studies under salinity (Zhang et al., 2012). Moreover, comparison of proteome responses in differentially-tolerant genotypes may help researchers to identify key proteins underlying the differences in stress tolerance.

The aim of this minireview is to summarize the major results obtained by proteomic studies in temperate cereal crops wheat and barley studied under abiotic and biotic stresses. Proteins affected by differential stress factors and proteins revealing a differential response between differentially-tolerant wheat and barley genotypes are discussed in a greater detail. Possibilities of utilization of proteins revealing a differential stress response between tolerant and sensitive genotypes as protein markers in breeding programs aimed at improvement of stress tolerance are suggested.

## Common features of stress response at proteome level

**Figure 1:**
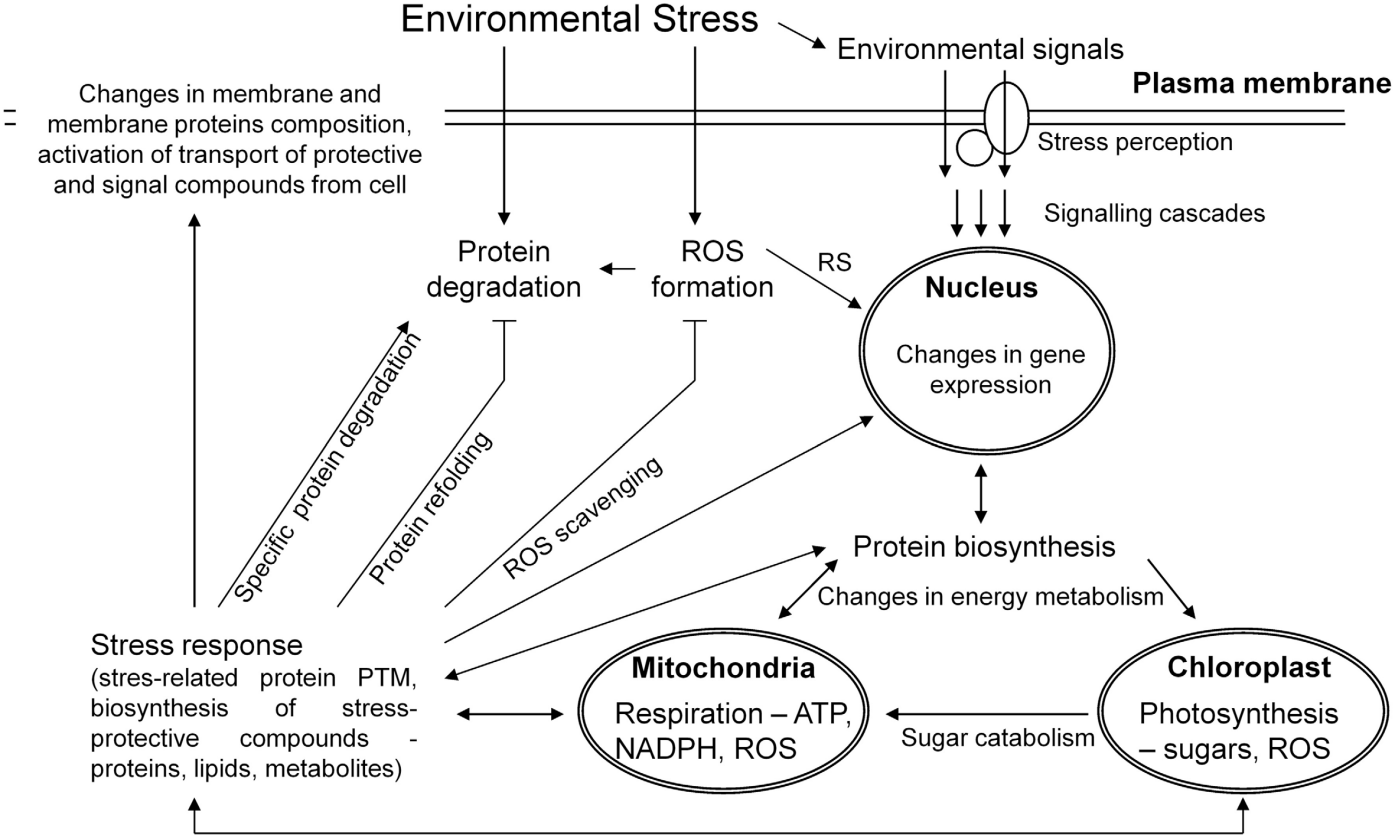
**A simplified scheme of plant cell response to an external stress stimulus leading to an activation of signaling cascades, changes in gene expression, activation of protein biosynthesis and degradation, profound changes in energy metabolism leading to an enhanced ATP biosynthesis and ROS production in chloroplasts and mitochondria resulting in ROS induced signaling (RS)**. Changes in protein biosynthesis lead to an enhanced production of both protein and non-protein (metabolite) stress protective compounds including ROS scavenging enzymes and metabolites, which participate in an active plant stress acclimation response including a feedback regulation of stress-induced signaling, gene, and protein expression mechanisms.

Plant stress response is a dynamic process aimed at an enhancement of plant stress tolerance and an establishment of a novel homeostasis between plant and environment (Figure 1). Several phases of plant stress response could be distinguished including an alarm phase, an acclimation phase, a resistance phase, an exhaustion phase when stress is too severe or lasts too long, and a recovery phase after a cessation of the given stress factor (Levitt, 1980; Larcher, 2003; Kosová et al., 2011). At proteome level, profound alterations in protein relative abundance were found between stressed and control plants as well as between differential genotypes (Table 1). During an alarm phase, stress induces profound alterations in proteins involved in cell signaling although these proteins are detected scarcely on 2DE gels due to their low abundance. An increase in 14-3-3 proteins as well as translationally controlled tumor protein homologs was detected in copper- and water-stressed wheat (Kang et al., 2012; Ghabooli et al., 2013; Li et al., 2013; Alvarez et al., 2014) and barley (Wendelboe-Nelson and Morris, 2012) and genotype-specific responses of β subunit of heterotrimeric G protein were found in salt-stressed wheat (Peng et al., 2009). Phosphorylation plays an important role in abiotic and biotic stress responses as shown on several kinases (calcium-dependent protein kinases CDPK, mitogen-activated protein kinases MAPK, sucrose non-fermenting-related kinases SnRK2), phosphatases (PP2C) and other signaling proteins (calmodulin 2-2) regulation (Yang et al., 2013; Zhang et al., 2014).

Stress acclimation represents an adaptive process aimed at an enhancement of plant stress tolerance. An active stress acclimation requires relatively high energy costs as indicated by profound alterations in energy metabolism. Practically all stresses induce an increased relative abundance of enzymes of carbohydrate catabolism such as glycolysis (glyceraldehyde-3-phosphate dehydrogenase GAPDH, triosephosphate isomerase TPI, enolase ENO), Krebs cycle (mitochondrial NAD^+^-dependent malate dehydrogenase (MDH; Vítámvás et al., 2012), aconitase (Jacoby et al., 2010, 2013; Budak et al., 2013) and components of mitochondrial ATP-synthase, namely β subunit of CF1 complex (Bahrman et al., 2004; Patterson et al., 2007; Vítámvás et al., 2012; Budak et al., 2013; Kosová et al., 2013b; Rollins et al., 2013; Xu et al., 2013) indicating an enhanced demand for energy. Regarding photosynthesis, an increase or a decrease in several photosynthetic proteins (proteins involved in primary photosynthetic reactions, carbon fixation, and Calvin cycle) have been observed depending on the severity of stress (Caruso et al., 2008, 2009; Ye et al., 2013). A downregulation of photosynthesis reactions under severe stress is reflected at proteome level by a decrease in D1 and D2 proteins in photosystem II reaction center (RC PSII), proteins of oxygen evolving complex (OEC), a decrease in chlorophyll *a*-*b* binding proteins in both photosystem (PS) I and II, a decrease in Fe-S complex in PSI, a downregulation of RubisCO and key Calvin cycle enzymes phosphoglycerate kinase and phosphoribulokinase in cold- and waterlogging-treated winter wheat (Li et al., 2014), salt-treated durum wheat (Caruso et al., 2008) and in drought-treated barley (Ghabooli et al., 2013) while an increase in OEC protein OEE2 was found in salt-treated barley (Rasoulnia et al., 2011; Fatehi et al., 2012). In addition, an increase in proteins with stimulating and protective functions such as RubisCO activase A (Bahrman et al., 2004; Caruso et al., 2008, 2009; Fatehi et al., 2012; Budak et al., 2013), a thermostable RubisCO activase B (Rollins et al., 2013), carbonic anhydrase (Caruso et al., 2008) and RubisCO large and small subunit binding proteins CPN60-α and CPN60-β was observed under various stresses (Caruso et al., 2008; Sarhadi et al., 2010; Kang et al., 2012; Budak et al., 2013; Kosová et al., 2013b; Xu et al., 2013).

An increased demand on energy under stress acclimation corresponds with a decreased abundance of enzymes (fructokinase-2, sucrose synthase-1) involved in biosynthesis of energy-rich compounds such as starch and a decrease in storage proteins (11S seed storage protein 2-like, legumin-like protein; Vítámvás et al., 2012; Kosová et al., 2013b).

Stress acclimation also reveals an enhanced demand on protein metabolism including both protein biosynthesis and degradation. Changes in the levels of eukaryotic translation initiation factors eIF3 subunit I, eIF5A2 (Kosová et al., 2013b), eIF4A (Rollins et al., 2013) and elongation factor eEF1-α (Budak et al., 2013), several ribosomal proteins, e.g., 60S proteins P0, P2A, L3, L38 (Fercha et al., 2014), or chloroplastic ribosomal proteins 30S-3, 50S-L12 (Ghabooli et al., 2013; Gharechahi et al., 2014), as well as in proteasome subunits such as 20S proteasome subunit α-type 1 and 6 (Rampitsch et al., 2006; Rinalducci et al., 2011a; Fercha et al., 2013; Ghabooli et al., 2013) and proteins of ubiquitin pathway involved in proteasome targeting such as ubiquitin conjugating enzyme E2 variant IC like (Kosová et al., 2013b) were reported indicating an enhanced protein turnover during stress acclimation.

Stress represents an enhanced risk of protein damage due to imbalances in cellular homeostasis. Therefore, increased abundances of several proteins with chaperone and other protective functions have been reported. Extreme temperatures, but also drought, pathogens, and other stresses cause an enhanced risk of protein misfolding and they are thus associated with an enhanced accumulation of chaperones from HSP superfamily, namely HSP70 (Rampitsch et al., 2006; Li et al., 2013; Rollins et al., 2013), HSP100 (Clp protease; Ashoub et al., 2013), and small HSPs (Skylas et al., 2002; Majoul et al., 2004; Hajheidari et al., 2007), but also others such as chopper chaperone (Vítámvás et al., 2012; Hlaváèková et al., 2013), serpins (Yang et al., 2010b; Fercha et al., 2013, 2014), and protein disulfide isomerase (Hajheidari et al., 2007; Vítámvás et al., 2012; Li et al., 2013). However, a decrease in HSP90 was reported under cold (Vítámvás et al., 2012). Disorders in cellular metabolism under stress lead to an enhanced risk of oxidative damage. At proteome level, an increased abundance of several reactive oxygen species (ROS) scavenging enzymes was found practically under each kind of stress (Hajheidari et al., 2007; Ford et al., 2011). Plants try to reduce a risk of ROS formation by several ways. The major one represents a downregulation of photosynthesis reactions which is associated with a decrease in D1 and D2 proteins in photosystem II reaction center (RC PSII), proteins of OEC, RubisCO small subunit and key Calvin cycle enzymes phosphoglycerate kinase, phosphoribulokinase and transketolase (Caruso et al., 2008; Ford et al., 2011; Ashoub et al., 2013). Other indirect ways how to reduce ROS lie in a reduced uptake of metal ions, especially iron, which can act as catalyzers of ROS formation. A reduced level of protein IDI2 and dioxygenases IDS2, IDS3 involved in iron uptake and phytosiderophore biosynthesis was found by Witzel et al. (2009) in salt-treated barley roots while an increased level of these enzymes was found by Patterson et al. (2007) in barley grown under elevated boron.

Several stresses including drought, heat, salinity, cold, but also mechanical wounding, induce an enhanced accumulation of proteins belonging to LEA superfamily. Late embryogenesis-abundant (LEA) superfamily includes at least five subclasses, the most important being LEA-II (dehydrins) and LEA-III proteins whose transcript and protein levels, and also phosphorylation level, have been reported to correlate with wheat and barley tolerance to low temperatures (Crosatti et al., 1995; Vágújfalvi et al., 2000, 2003; Vítámvás et al., 2007; Kosová et al., 2008c, 2013c; Sarhadi et al., 2010), drought (Labhilili et al., 1995; Brini et al., 2007) and other stresses.

Several stresses, especially biotic ones, are associated with an induction of protective proteins from PR superfamily. Pathogenesis-related (PR) proteins encompass 16 groups involved in defense against microbial and fungal pathogens (Edreva, 2005). Many of PR proteins can resist acidic pH, they reveal enzymatic activities aimed at modifications of cell wall, and ROS scavenging functions (some germins and germin-like proteins reveal manganese superoxide dismutase (Mn-SOD) and oxalate oxidase activities). An enhanced abundance of several PR proteins was reported not only in cereals exposed to fungal pathogens such as *Fusarium* (class-II chitinase, β-amylase, thaumatin-like protein, PR9–peroxidase; Yang et al., 2010a,b; Eggert and Pawelzik, 2011; Eggert et al., 2011), but also under abiotic stresses such as cold (β-1,3-glucanase, chitinase, PR4, thaumatin-like protein; Sarhadi et al., 2010; Kosová et al., 2013b; Gharechahi et al., 2014), salinity (germin-like protein, PR10; Fatehi et al., 2012; Kamal et al., 2012; Witzel et al., 2014), and others.

Stresses also affect other aspects of cellular metabolism. An increased abundance of methionine synthase catalyzing formation of methionine or S-adenosylmethionine synthase (SAMS) catalyzing formation of S-adenosylmethionine (SAM) has been reported (Bahrman et al., 2004; Patterson et al., 2007; Witzel et al., 2009; Vítámvás et al., 2012; Kosová et al., 2013b; Xu et al., 2013). SAM represents not only a universal methyl donor in regulation of DNA heterochromatin formation and gene expression, but it is also a precursor of several stress-related metabolites as glycine betaine, polyamines, hydroxymugineic acids (phytosiderophore precursors; Mori and Nishizawa, 1987) and ethylene. Alterations in glutamine synthetase (GS) have been reported under drought (Ford et al., 2011; Kang et al., 2012) and cold (Hlaváèková et al., 2013) indicating an important role of nitrogen assimilation and proline biosynthesis in stress acclimation.

Stress affects cellular transport and membrane properties. An enhanced need for ion transport and thus an associated increase in plasma membrane and tonoplast ion transporters such as V-ATPase has been reported not only under salinity (Peng et al., 2009), but also under other stresses such as drought (Ghabooli et al., 2013), heat (Majoul et al., 2004) and osmotic stress (Ye et al., 2013; Zhang et al., 2014). Differential phosphorylation of several transport proteins such as aquaporins, H^+^-ATPase or monosaccharide sensing protein 2, was also reported in response to stress (Zhang et al., 2014). The effect of several stresses on cell wall remodeling is indicated by alterations in several enzymes involved in lignin metabolism such as caffeoyl-coenzyme A O-methyltransferase CCOMT indicating an important role of cell wall in plant stress response (Sugimoto and Takeda, 2009; Ghabooli et al., 2013).

Long-term and regularly occurring stress factors such as cold during winter also affect plant development. At proteome level, significant changes in the level of small glycine-rich RNA-binding proteins (sGRPs) and in lectins, glycoproteins involved in saccharide signaling, were found in wheat (Rinalducci et al., 2011b; Kosová et al., 2013b). Ricin B lectin 2 was reported to be induced by cold in crowns of both winter barley (Hlaváèková et al., 2013) and winter wheat (Kosová et al., 2013b). Lectin VER2 was reported to accumulate in winter wheat shoot apex until vernalization (Yong et al., 2003; Rinalducci et al., 2011b). Differences in sGRPs and VER2 levels between spring and winter wheat growth habits indicate a differential response to cold within wheat germplasm (Kosová et al., 2013b).

## Proteins revealing a differential response between stress-tolerant and stress-sensitive genotypes

A differential ability of various wheat and barley genotypes to cope with several stresses is reflected also at protein level. Stress-tolerant genotypes do not suffer from a disruption of energy metabolism when exposed to moderate stress levels; moreover, when exposed to stress, they can increase an abundance of key enzymes of energy metabolism to increase ATP production as indicated by a differential response observed in several photosynthesis-related proteins (RubisCO subunits, RubisCO activase), ROS scavenging enzymes as well as respiration (Krebs cycle) enzymes. Quantitative differences in Krebs cycle enzymes such as mitochondrial NAD^+^-dependent MDH between two differentially frost-tolerant winter wheats (Vítámvás et al., 2012), in aconitase (Budak et al., 2013), thioredoxin *h* and glutathione-S-transferase (GST; Hajheidari et al., 2007; Sarhadi et al., 2010), lipoxygenase 1 and 2 (Alvarez et al., 2014) between differentially drought-tolerant wheats; in Cu/Zn-SOD, Mn-SOD (Ford et al., 2011; Xu et al., 2013), glyoxysomal MDH (gMDH; Ashoub et al., 2013), GST (Rasoulnia et al., 2011), class III peroxidase, catalase and lipoxygenase (Wendelboe-Nelson and Morris, 2012) between differentially drought- and salt-tolerant barleys; in Mn-SOD, MDH and aconitase between salt-treated wheat and wheat × *Lophopyrum elongatum* amphiploid (Jacoby et al., 2013), and a downregulation of MDH and isocitrate dehydrogenase in cold-sensitive spring wheat (Rinalducci et al., 2011a) indicate a crucial role of mitochondrial respiration and ROS metabolism in stress acclimation. Along with these data, a differential abundance in storage proteins such as legumin-like protein between two differentially frost-tolerant winter wheats was found by Vítámvás et al. (2012) indicating a higher demand on energy ensured by storage compound degradation in the less-tolerant genotype. Moreover, tolerant genotypes can also afford to accumulate higher amounts of stress-protective proteins such as PR proteins (Witzel et al., 2014) and ABA-responsive proteins (Alvarez et al., 2014). A significant correlation between wheat WCS120 and barley DHN5 dehydrin relative accumulation and acquired frost tolerance (FT) determined as lethal temperature for 50 % of the sample (LT50) was reported for winter genotypes grown under both cold and moderate cold temperatures (Vítámvás et al., 2007, 2010; Kosová et al., 2008c, 2013c). WCS120 and DHN5 can be thus considered promising FT markers.

Stress-tolerant and stress-sensitive genotypes or related plant species also reveal significant differences in proteins involved in regulation of cell cycle and plant development. Factor eIF5A2 does not only regulate translation inititation, but it is also known to participate in the regulation of cell cycle switch between cell proliferation and death (Thompson et al., 2004). Under salinity, a decreased abundance of eIF5A2 with respect to control was found in both salt-sensitive common wheat and salt-tolerant *T. aestivum* × *Thinopyrum ponticum* hybrid (Wang et al., 2008); however, a decrease in *T. aestivum* × *Th. ponticum* was much lower than in *T. aestivum* indicating a higher cell proliferation rate in the salt-tolerant hybrid. A differential abundance in lectin VER2 between cold-treated spring and winter wheat cultivars corresponds to a differential developmental response with a winter wheat revealing a developmental arrest while a spring wheat revealing a progression to reproductive phase as indicated at proteome and phytohormone levels (Kosová et al., 2012, 2013b).

## Conclusions and future perspectives

Both abiotic and biotic stress factors induce an active plant stress response including a profound reorganization of plant proteome. Comparative proteomic studies are usually carried out on a limited range of plant material due to their expensiveness and much of sophisticated work. However, they can significantly contribute to identification of novel proteins revealing a differential response in abundance or PTMs between differentially-tolerant genotypes and representing potential protein markers of stress tolerance. The potential markers should be tested on a broad range of genotypes using simple protein quantification methods as ELISA or immunoblots which can be utilized by breeders. As an example, proteomic studies on cold-treated winter wheats resulting in an identification and testing of dehydrin proteins as FT markers can be given (Vítámvás et al., 2007, 2010). Recent publication of draft barley (The International Barley Genome Sequencing Consortium, 2012) and wheat (The International Wheat Genome Sequencing Consortium, 2014) genome sequences will significantly contribute to protein identification, sequentional characterization and preparation of specific antibodies which will stimulate further research and applications in breeding for an improved stress tolerance.

## Author contributions

Klára Kosová has outlined the idea and prepared the text. Pavel Vítámvás and Ilja T. Prášil contributed to preparation, drafting, critical reading, and publication of the manuscript.

### Conflict of interest statement

The authors declare that the research was conducted in the absence of any commercial or financial relationships that could be construed as a potential conflict of interest.

## References

[B1] Alvarez S. Choudhury S. R. Pandey S. (2014). Comparative quantitative proteomics analysis of the ABA response of roots of drought-sensitive and drought-tolerant wheat varieties identifies proteomic signatures of drought adaptability. J. Proteome Res. 13, 1688–1701. 10.1021/pr401165b 24475748

[B2] Ashoub A. Beckhaus T. Berberich T. Karas M. Brüggemann W. (2013). Comparative analysis of barley leaf proteome as affected by drought stress. Planta 237, 771–781. 10.1007/s00425-012-1798-4 23129216

[B3] Bahrman N. Le Gouis J. Negroni L. Amilhat L. Leroy P. Lainé A. L.. (2004). Differential protein expression assessed by two-dimensional gel electrophoresis for two wheat varieties grown at four nitrogen levels. Proteomics 4, 709–719. 10.1002/pmic.200300571 14997493

[B4] Brini F. Hanin M. Lumbreras V. Irar S. Pagès M. Masmoudi K. (2007). Functional characterization of DHN-5, a dehydrin showing a differential phosphorylation pattern in two Tunisian durum wheat (*Triticum durum* Desf.) varieties with marked differences in salt and drought tolerance. Plant Sci. 172, 20–28 10.1016/j.plantsci.2006.07.011

[B5] Budak H. Akpinar B. A. Unver T. Turktas M. (2013). Proteome changes in wild and modern wheat leaves upon drought stress by two-dimensional electrophoresis and nanoLC-ESI-MS/MS. Plant Mol. Biol. 83, 89–103. 10.1007/s11103-013-0024-5 23443681

[B6] Caruso G. Cavaliere C. Foglia P. Gubbiotti R. Samperi R. Laganà A. (2009). Analysis of drought responsive proteins in wheat (*Triticum durum*) by 2D-PAGE and MALDI-TOF mass spectrometry. Plant Sci. 177, 570–576 10.1016/j.plantsci.2009.08.007

[B7] Caruso G. Cavaliere C. Guarino C. Gubbiotti R. Foglia P. Laganà A. (2008). Identification of changes in *Triticum durum* L. leaf proteome in response to salt stress by two-dimensional electrophoresis and MALDI-TOF mass spectrometry. Anal. Bioanal. Chem. 391, 381–390. 10.1007/s00216-008-2008-x 18365183

[B8] Cattivelli L. Rizza F. Badeck F. W. Mazzucotelli E. Mastrangelo A. M. Francia E. (2008). Drought tolerance improvement in crop plants: An integrated view from breeding to genomics. Field Crops Res. 105, 1–14 10.1016/j.fcr.2007.07.004

[B9] Crosatti C. Soncini C. Stanca A. M. Cattivelli L. (1995). The accumualtion of a cold-regulated chloroplastic protein is light dependent. Planta 195, 458–463. 7647681 10.1007/BF00203644

[B10] Edreva A. (2005). Pathogenesis-related proteins: research progress in the last 15 years. Gen. Appl. Plant Physiol. 31, 105–124.

[B11] Eggert K. Pawelzik E. (2011). Proteome analysis of Fusarium head blight in grains of naked barley (*Hordeum vulgare* subsp. nudum). Proteomics 11, 972–985. 10.1002/pmic.201000322 21271677

[B12] Eggert K. Zörb C. Mühling K. H. Pawelzik E. (2011). Proteome analysis of *Fusarium* infection in emmer grains (*Triticum dicoccum*). Plant Pathol. 60, 918–928 10.1111/j.1365-3059.2011.02442.x

[B13] Fatehi F. Hosseinzadeh A. Alizadeh H. Brimavandi T. Struik P. C. (2012). The proteome response of salt-resistant and salt-sensitive barley genotypes to long-term salinity stress. Mol. Biol. Rep. 39, 6387–6397. 10.1007/s11033-012-1460-z 22297690

[B14] Fercha A. Capriotti A. L. Caruso G. Cavaliere C. Gherroucha H. Samperi R.. (2013). Gel-free proteomics reveal potential biomarkers of priming-induced salt tolerance in durum wheat. J. Proteomics 91, 486–499. 10.1016/j.jprot.2013.08.010 23973468

[B15] Fercha A. Capriotti A. L. Caruso G. Cavaliere C. Samperi R. Stampachiacchiere S.. (2014). Comparative analysis of metabolic proteome variation in ascorbate-primed and unprimed wheat seeds during germination under salt stress. J. Proteomics 108, 238–257. 10.1016/j.jprot.2014.04.040 24859728

[B16] Ford K. L. Cassin A. Bacic A. (2011). Quantitative proteomic analysis of wheat cultivars with differing drought stress tolerance. Front. Plant. Sci. 2:44. 10.3389/fpls.2011.00044 22639595 PMC3355674

[B17] Ghabooli M. Khatabi B. Ahmadi F. S. Sepehri M. Mizraei M. Amirkhani A.. (2013). Proteomics study reveals the molecular mechanisms underlying water stress tolerance induced by *Piriformospora indica* in barley. J. Proteomics 94, 289–301. 10.1016/j.jprot.2013.09.017 24120527

[B18] Gharechahi J. Alizadeh H. Reza Naghavi M. Sharifi G. (2014). A proteomic analysis to identify cold acclimation associated proteins in wild wheat (*Triticum urartu* L.). Mol. Biol. Rep. 41, 3897–3905. 10.1007/s11033-014-3257-8 24535272

[B19] Gonzalez-Fernandez R. Jorrin-Novo J. V. (2012). Contribution of proteomics to the study of plant pathogenic fungi. J. Proteome Res. 11, 3–16. 10.1021/pr200873p 22085090

[B20] Hajheidari M. Eivazi A. Buchanan B. B. Wong J. H. Majidi I. Salekdeh G. H. (2007). Proteomics uncovers a role for redox in drought tolerance in wheat. J. Proteome Res. 6, 1451–1460. 10.1021/pr060570j 17343403

[B21] Hlaváèková I. Vítámvás P. Šantrůček J. Kosová K. Zelenková S. Prášil I. T.. (2013). Proteins involved in distinct phases of cold hardening process in frost resistant winter barley (*Hordeum vulgare* L.) cv. Luxor. Int. J. Mol. Sci. 44, 8000–8024. 10.3390/ijms14048000 23584021 PMC3645728

[B22] Hossain Z. Nouri M. Z. Komatsu S. (2012). Plant cell organelle proteomics in response to abiotic stress. J. Proteome Res. 11, 37–48. 10.1021/pr200863r 22029473

[B23] Jacoby R. P. Millar A. H. Taylor N. L. (2010). Wheat mitochondrial proteomes provide new links between antioxidant defense and plant salinity tolerance. J. Proteome Res. 9, 6595–6604. 10.1021/pr1007834 21043471

[B24] Jacoby R. P. Millar A. H. Taylor N. L. (2013). Investigating the role of respiration in plant salinity tolerance by analyzing mitochondrial proteomes from wheat and a salinity-tolerant amphiploid (Wheat × *Lophopyrum elongatum*). J. Proteome Res. 12, 4807–4829. 10.1021/pr400504a 23895732

[B25] Jorrin-Novo J. V. Maldonado A. M. Echavarría-Zomeno S. Valledor L. Castillejo M. A. Curto M.. (2009). Plant proteomics update (2007–2008): second-generation proteomic techniques, an appropriate experimental design, and data analysis to fulfill MIAPE standards, increase plant proteome coverage and expand biological knowledge. J. Proteomics 72, 285–314. 10.1016/j.jprot.2009.01.026 19367730

[B26] Kamal A. H. M. Cho K. Kim D. E. Uozumi N. Chung K. Y. Lee S. Y.. (2012). Changes in physiology and protein abundance in salt-stressed wheat chloroplasts. Mol. Biol. Rep. 39, 9059–9074. 10.1007/s11033-012-1777-7 22736107

[B27] Kang G. Li G. Xu W. Peng X. Han Q. Zhu Y.. (2012). Proteomics reveals the effects of salicylic acid on growth and tolerance to subsequent drought stress in wheat. J. Proteome Res. 11, 6066–6079. 10.1021/pr300728y 23101459

[B28] Kosová K. Chrpová J. Šíp V. (2008b). Recent advances in breeding of cereals for resistance to barley yellow Dwarf virus - a review. Czech J. Genet. Plant Breed. 44, 1–10.

[B29] Kosová K. Chrpová J. Šíp V. (2009). Cereal resistance to Fusarium head blight and possibilities of its improvement through breeding. Czech J. Genet. Plant Breed. 45, 87–105.

[B30] Kosová K. Holková L. Prášil I. T. Prášilová P. Bradáčová M. Vítámvás P.. (2008c). Expression of dehydrin 5 during the development of frost tolerance in barley (*Hordeum vulgare*). J. Plant Physiol. 165, 1142–1151. 10.1016/j.jplph.2007.10.009 18242771

[B31] Kosová K. Prášil I. T. Vítámvás P. (2008a). The relationship between vernalization- and photoperiodically-regulated genes and the development of frost tolerance in wheat and barley. Biol. Plant. 52, 601–615 10.1007/s10535-008-0120-6

[B32] Kosová K. Prášil I. T. Vítámvás P. (2013a). Protein contribution to plant salinity response and tolerance acquisition. Int. J. Mol. Sci. 14, 6757–6789. 10.3390/ijms14046757 23531537 PMC3645664

[B33] Kosová K. Prášil I. T. Vítámvás P. Dobrev P. Motyka V. Floková K.. (2012). Complex phytohormone responses during the cold acclimation of two wheat cultivars differing in cold tolerance, winter Samanta and spring Sandra. J. Plant Physiol. 169, 567–576. 10.1016/j.jplph.2011.12.013 22304971

[B34] Kosová K. Vítámvás P. Planchon S. Renaut J. Vanková R. Prášil I. T. (2013b). Proteome analysis of cold response in spring and winter wheat (*Triticum aestivum*) crowns reveals similarities in stress adaptation and differences in regulatory processes between the growth habits. J. Proteome Res. 12, 4830–4845. 10.1021/pr400600g 24047233

[B35] Kosová K. Vítámvás P. Prášil I. T. Renaut J. (2011). Plant proteome changes under abiotic stress—contribution of proteomics studies to understanding plant stress response. J. Proteomics 74, 1301–1322. 10.1016/j.jprot.2011.02.006 21329772

[B36] Kosová K. Vítámvás P. Prášilová P. Prášil I. T. (2013c). Accumulation of WCS120 and DHN5 proteins in differently frost-tolerant wheat and barley cultivars grown under a broad temperature scale. Biol. Plant. 57, 105–112 10.1007/s10535-012-0237-5

[B37] Labhilili M. Joudrier P. Gautier M. F. (1995). Characterization of cDNAs encoding *Triticum durum* dehydrins and their expression patterns in cultivars that differ in drought tolerance. Plant Sci. 112, 219–230 10.1016/0168-9452(95)04267-9

[B38] Larcher W. (2003). Physiological Plant Ecology, 4th Edn. Berlin; Heidelberg; Springer Verlag.

[B39] Levitt J. (1980). Responses of Plants to Environmental Stress. Chilling, Freezing and High Temperature Stresses, 2nd Edn. New York, NY: Academic Press.

[B40] Li G. Peng X. Xuan H. Wei L. Yang Y. Guo T.. (2013). Proteomic analysis of leaves and roots of common wheat (*Triticum aestivum* L.) under copper-stress conditions. J. Proteome Res. 12, 4846–4861. 10.1021/pr4008283 24074260

[B41] Li X. Cai J. Liu F. Dai T. Cao W. Jiang D. (2014). Physiological, proteomic and transcriptional responses of wheat to combination of drought or waterlogging with late spring low temperature. Funct. Plant Biol. 41, 690–703 10.1071/FP13306 32481024

[B42] Majoul T. Bancel E. Triboi E. Ben Hamida J. Branlard G. (2004). Proteomic analysis of the effect of heat stress on hexaploid wheat grain: Characterization of heat-responsive proteins from non-prolamins fraction. Proteomics 4, 505–513. 10.1002/pmic.200300570 14760723

[B43] Mittler R. (2006). Abiotic stress, the field environment and stress combination. Trends Plant Sci. 11, 15–19. 10.1016/j.tplants.2005.11.002 16359910

[B44] Mori S. Nishizawa N. (1987). Methionine as a dominant precursor of phytosiderophores in *Graminaceae* plants. Plant Cell Physiol. 28, 1081–1092.

[B45] Munns R. (2005). Genes and salt tolerance: bringing them together. New Phytol. 167, 645–663. 10.1111/j.1469-8137.2005.01487.x 16101905

[B46] Patterson J. Ford K. Cassin A. Natera S. Bacic A. (2007). Increased abundance of proteins involved in phytosiderophore production in boron-tolerant barley. Plant Physiol. 144, 1612–1631. 10.1104/pp.107.096388 17478636 PMC1914127

[B47] Peng Z. Wang M. Li F. Lv H. Li C. Xia G. (2009). A proteomic study of the response to salinity and drought stress in an introgression strain of bread wheat. Mol. Cell. Proteomics 8, 2676–2686. 10.1074/mcp.M900052-MCP200 19734139 PMC2816014

[B48] Rampitsch C. Bykova N. V. McCallum B. Beimcik E. Ens W. (2006). Analysis of the wheat and *Puccinia triticina* (leaf rust) proteomes during a susceptible host-pathogen interaction. Proteomics 6, 1897–1907. 10.1002/pmic.200500351 16479535

[B49] Rasoulnia A. Bihamta M. R. Peyghambari S. A. Alizadeh H. Rahnama A. (2011). Proteomic response of barley leaves to salinity. Mol. Biol. Rep. 38, 5055–5063. 10.1007/s11033-010-0651-8 21181273

[B50] Rinalducci S. Egidi K. G. Mahfoozi S. Godehkahriz S. J. Zolla L. (2011b). The influence of temperature on plant development in a vernalization-requiring winter wheat: A 2-DE based proteomic investigation. J. Proteomics 74, 643–659. 10.1016/j.jprot.2011.02.005 21320650

[B51] Rinalducci S. Egidi M. G. Karimzadeh G. Jazii F. Zolla L. (2011a). Proteomic analysis of a spring wheat cultivar in response to prolonged cold stress. Electrophoresis 32, 1807–1818. 10.1002/elps.201000663 21710550

[B52] Rollins J. A. Habte E. Templer S. E. Colby T. Schmidt J. von Korff M. (2013). Leaf proteome alterations in the context of physiological and morphological responses to drought and heat stress in barley (*Hordeum vulgare* L.). J. Exp. Bot. 64, 3201–3212. 10.1093/jxb/ert158 23918963 PMC3733145

[B53] Sarhadi E. Mahfoozi S. Hosseini S. A. Salekdeh G. H. (2010). Cold accliamtion proteome analysis reveals close link between upregulation of low-temperature associated proteins and vernalization fulfillment. J. Proteome Res. 9, 5658–5667. 10.1021/pr100475r 20804221

[B56] Sergeant K. Renaut J. (2010). Plant biotic stress and proteomics. Curr. Proteomics 7, 275–297 10.2174/157016410793611765

[B57] Skylas D. J. Cordwell S. J. Hains P. G. Larsen M. R. Basseal D. J. Walsch B. J. (2002). Heat shock of wheat during grain filling: Proteins associated with heat tolerance. J. Cereal Sci. 35, 175–188 10.1006/jcrs.2001.0410

[B58] Sugimoto M. Takeda K. (2009). Proteomic analysis of specific proteins in the root of salt-tolerant barley. Biosci. Biotechnol. Biochem. 73, 2762–2765. 10.1271/bbb.90456 19966459

[B54] The International Barley Genome Sequencing Consortium. (2012). A physical, genetic and functional sequence assembly of the barley genome. Nature 491, 711–717. 10.1038/nature11543 23075845

[B55] The International Wheat Genome Sequencing Consortium. (2014). Slicing the wheat genome. Science 345, 285–287. 10.1126/science25035484

[B59] Thomashow M. F. (1999). Plant cold acclimation. Freezing tolerance genes and regulatory mechanisms. Annu. Rev. Plant Physiol. Plant Mol. Biol. 50, 571–599. 10.1146/annurev.arplant.50.1.571 15012220

[B60] Thompson J. E. Hopkins M. E. Taylor C. Wang T. W. (2004). Regulation of senescence by eukaryotic translation initiation factor 5A: implications for plant growth and development. Trends Plant Sci. 9, 174–179. 10.1016/j.tplants.2004.02.008 15063867

[B61] Vágújfalvi A. Crosatti C. Galiba G. Dubcovsky J. Cattivelli L. (2000). Two loci of chromosome 5A regulate the differential cold-dependent expression of the *cor-14b* gene in frost-tolerant and frost-sensitive genotypes. Mol. Gen. Genet. 263, 194–200. 10.1007/s004380051160 10778737

[B62] Vágújfalvi A. Galiba G. Cattivelli L. Dubcovsky J. (2003). The cold-regulated transcriptional activator *Cbf3* is linked to the frost-tolerance locus *Fr-A2* on wheat chromosome 5A. Mol. Gen. Genomics 269, 60–67. 10.1007/s00438-003-0806-6 12715154 PMC4743881

[B63] Vítámvás P. Kosová K. Prášilová P. Prášil I. T. (2010). Accumulation of WCS120 protein in wheat cultivars grown at 9°C or 17°C in relation to their winter survival. Plant Breed. 129, 611–616. 10.1111/j.1439-0523.2010.01783.x19819243

[B64] Vítámvás P. Prášil I. T. Kosová K. Planchon S. Renaut J. (2012). Analysis of proteome and frost tolerance in chromosome 5A and 5B reciprocal substitution lines between two winter wheats during long-term cold acclimation. Proteomics 12, 68–85. 10.1002/pmic.201000779 22065556

[B65] Vítámvás P. Saalbach G. Prášil I. T. Čapková V. Opatrná J. Jahoor A. (2007). WCS120 protein family and proteins soluble upon boiling in cold-acclimated winter wheat. J. Plant Physiol. 164, 1197–1207. 10.1016/j.jplph.2006.06.011 16963156

[B66] Wang M. C. Peng Z. Y. Li C. L. Li F. Liu C. Xia G. M. (2008). Proteomic analysis on a high salt tolerance introgression strain of *Triticum aestivum*/*Thinopyrum ponticum*. Proteomics 8, 1470–1489. 10.1002/pmic.200700569 18383010

[B67] Wendelboe-Nelson C. Morris P. C. (2012). Proteins linked to drought tolerance revealed by DIGE analysis of drought resistant and susceptible barley varieties. Proteomics 12, 3374–3385. 10.1002/pmic.201200154 23001927

[B68] Witzel K. Matros A. Strickert M. Kaspar S. Peukert M. Mühling K. H.. (2014). Salinity stress in roots of contrasting barley genotypes reveals time-distinct and genotype-specific patterns for defined proteins. Mol. Plant 7, 336–355. 10.1093/mp/sst063 24004485

[B69] Witzel K. Weidner A. Surabhi G. K. Börner A. Mock H. P. (2009). Salt stress-induced alterations in the root proteome of barley genotypes with contrasting response towards salinity. J. Exp. Bot. 60, 3545–3557. 10.1093/jxb/erp198 19671579 PMC2724703

[B70] Xu J. Li Y. Sun J. Du L. Zhang Y. Yu Q.. (2013). Comparative physiological and proteomic response to abrupt low temperature stress in two winter wheat cultivars differing in low temperature tolerance. Plant Biol. 15, 292–303. 10.1111/j.1438-8677.2012.00639.x 22963252

[B71] Yang F. Jensen J. D. Spliid N. H. Svensson B. Jacobsen S. Jørgensen L. N.. (2010b). Investigation of the effect of nitrogen on severity of Fusarium Head Blight in barley. J. Proteomics 73, 743–752. 10.1016/j.jprot.2009 19895910

[B72] Yang F. Jensen J. D. Svensson B. Jørgensen H. J. L. Collinge D. B. Finnie C. (2010a). Analysis of early events in the interaction between *Fusarium graminearum* and the susceptible barley (*Hordeum vulgare*) cultivar Scarlett. Proteomics 10, 3748–3755. 10.1002/pmic.201000243 20925056

[B73] Yang F. Melo-Braga M. N. Larsen M. R. Jørgensen H. J. L. Palmisano G. (2013). Battle through signaling between wheat and the fungal pathogen *Septoria tritici* revealed by proteomics and phosphoproteomics. Mol. Cell. Proteomics 12, 2497–2508. 10.1074/mcp.M113.027532 23722186 PMC3769326

[B74] Ye J. Wang S. Zhang F. Xie D. Yao Y. (2013). Proteomic analysis of leaves of different wheat genotypes subjected to PEG6000 stress and rewatering. Plant OMICS J. 6, 286–294.

[B75] Yong W. D. Xu Y. Y. Xu W. Z. Wang X. Li N. Wu J. S.. (2003). Vernalization-induced flowering in wheat is mediated by a lectin-like gene *VER2*. Planta 217, 261–270. 10.1007/s00425-003-0994-7 12783334

[B76] Zhang H. Han B. Wang T. Chen S. Li H. Zhang Y.. (2012). Mechanisms of plant stress response: Insights from proteomics. J. Proteome Res. 11, 49–67. 10.1021/pr200861w 22017755

[B77] Zhang M. Lv D. Ge P. Bian Y. Chen G. Zhu G.. (2014). Phosphoproteome analysis reveals new drought response and defense mechanisms of seedling leaves in bread wheat (*Triticum aestivum* L.). J. Proteomics 109, 290–308. 10.1016/j.jprot.2014.07.010 25065648

